# Transactions of the American Medical Association

**Published:** 1877-11-20

**Authors:** 


### BOOK NOTICES.

Transactions of the American Medical Association. Prize essay. Supplement to volume 27, 1876. (Phil. Collins, Printer, 705 Jayne street.

This is a large volume of over 600 pages, constituting the prize essay of H. Culbertson, M. D. professor of Opthalmology in the Columbus Medical College, etc. Contains tabular statements of operations for excesion of the larger joints of the extremities, with illustrations, etc. The work is valuable, and evinces great labor, skill and diligence on the part of the writer.

